# *Notes from the Field*: Update on Excess Deaths Associated with the COVID-19 Pandemic — United States, January 26, 2020–February 27, 2021

**DOI:** 10.15585/mmwr.mm7015a4

**Published:** 2021-04-16

**Authors:** Lauren M. Rossen, Amy M. Branum, Farida B. Ahmad, Paul D. Sutton, Robert N. Anderson

**Affiliations:** 1 National Center for Health Statistics, CDC.

Estimates of excess deaths, defined as the number of persons who have died from all causes, above the expected number of deaths for a given place and time, can provide a comprehensive account of mortality likely related to the COVID-19 pandemic, including deaths that are both directly and indirectly associated with COVID-19. Since April 2020, CDC’s National Center for Health Statistics (NCHS) has published weekly data on excess deaths associated with the COVID-19 pandemic (*1*). A previous report identified nearly 300,000 excess deaths during January 26–October 3, 2020, with two thirds directly associated with COVID-19 (*2*). Using more recent data from the National Vital Statistics System (NVSS), CDC estimated that 545,600–660,200 excess deaths occurred in the United States during January 26, 2020–February 27, 2021.

Using weekly historical and provisional NVSS mortality data from 2013 through February 27, 2021, expected numbers of deaths were estimated using overdispersed Poisson regression models with spline terms to account for seasonal patterns (*1*,*2*). The average expected number, as well as the upper bound of the 95% prediction interval, were used as thresholds to determine the number of excess deaths.* Observed numbers of deaths were weighted to account for incomplete reporting by jurisdictions (50 states and the District of Columbia), primarily in the most recent 8 weeks, where the weights were estimated based on completeness of provisional data during the past year (*1*). Weekly NVSS data on excess deaths occurring from January 26 (the week ending February 1, 2020) through February 27, 2021, were then examined to quantify the number of excess deaths from all causes and the number of deaths from all causes other than COVID-19.^†^

During January 26, 2020–February 27, 2021, an estimated 545,600–660,200 more persons than expected died in the United States from all causes (Figure). The estimated number of excess deaths peaked during the weeks ending April 11, 2020, August 1, 2020, and January 2, 2021. Approximately 75%–88% of excess deaths were directly associated with COVID-19. Excluding deaths directly associated with COVID-19, an estimated 63,700–162,400 more persons than expected died from other causes.

**FIGURE:**
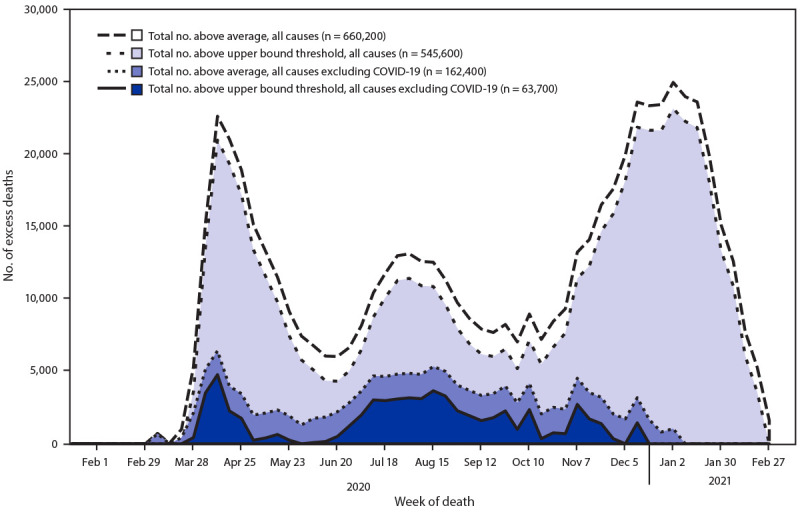
Weekly and total numbers of excess deaths from all causes, and from all causes other than COVID-19* above the average number expected and the upper bound of the 95% prediction interval^†^ — United States, January 26, 2020–February 27, 2021 * Weekly numbers of deaths from all causes and from all causes other than COVID-19 were obtained from the National Vital Statistics System. ^†^ The average expected number and the upper bound of the 95% prediction interval (the range of values likely to contain the value of a single new observation) were estimated using overdispersed Poisson regression models of 2013 mortality data to the most recent week, with spline terms to account for seasonal patterns. The numbers of excess deaths correspond to the observed numbers of deaths above each threshold. Total numbers of excess deaths were summed from January 26, 2020, through February 27, 2021.

Estimates of excess deaths provide insight into the impact of the COVID-19 pandemic beyond tracking data on the numbers of deaths directly associated with COVID-19.^§^ Data on reported COVID-19 deaths might be limited by factors such as the availability and use of diagnostic testing and the accurate and complete reporting of cause-of-death information on the death certificate (*3*). Excess death analyses are not subject to these limitations because they examine historical trends in all-cause mortality to determine the degree to which observed numbers of deaths differ from historical trends.

The findings in this report are subject to at least three limitations. First, because of reporting lags, estimated numbers of deaths in the most recent weeks are likely underestimated and might increase as more data become available.^¶^ Second, different methods for estimating the expected numbers of deaths might lead to different results, and the models employed for this report might not fully account for population growth or aging. Another report on provisional 2020 mortality data, which described annual mortality rates by demographic factors and leading causes of death, but did not examine trends in excess deaths, found that age-adjusted death rates, which do account for population growth and aging, increased by 15.9% from 2019 to 2020 (*3*). Finally, estimates of excess deaths not associated with COVID-19 might represent misclassified COVID-19 deaths or deaths indirectly associated with the pandemic (e.g., because of disruptions in health care access or utilization). For example, a previous report described declines in emergency department visits for heart attack, stroke, and hyperglycemic crisis in early 2020 (*4*). The excess death analyses presented here cannot distinguish between excess deaths that might have been misclassified COVID-19 deaths or those that might have been indirectly associated with the pandemic.

These updated estimates indicate that approximately one half to two thirds of one million excess deaths occurred during January 26, 2020–February 27, 2021, suggesting that the overall impact of the COVID-19 pandemic on mortality is substantially greater than the number of COVID-19 deaths. These data can help guide efforts to prevent infection and mortality directly or indirectly associated with COVID-19. CDC’s NCHS continues to provide weekly data on excess deaths (*1*) to enable near real-time tracking of mortality associated with the COVID-19 pandemic.

## Footnotes

* The average expected number and the upper bound of the 95% prediction interval (the range of values likely to contain the value of a single new observation, given what has already been observed) were used to determine the number of excess deaths or observed numbers above each threshold.

† Deaths from all causes excluding COVID-19 were calculated by subtracting the number of confirmed or presumed COVID-19 deaths from the total number of deaths. Deaths with confirmed or presumed COVID-19 were assigned the *International Classification of Diseases, Tenth Revision* code U07.1 as a contributing or underlying cause of death on the death certificate.

§
https://covid.cdc.gov/covid-data-tracker/#trends_dailytrendsdeaths

¶ Data are provisional and subject to change. Data during the most recent 8 weeks are incomplete because of the lag in time between the death and the completion of the death certificate, submission to NCHS, and processing of the data for reporting purposes. This delay can range from 1 week to 8 weeks or more depending on the jurisdiction and cause of death.
